# Vibration-Assisted Synthesis of Nanoporous Anodic Aluminum Oxide (AAO) Membranes

**DOI:** 10.3390/mi13122236

**Published:** 2022-12-16

**Authors:** Urte Cigane, Arvydas Palevicius, Giedrius Janusas

**Affiliations:** Faculty of Mechanical Engineering and Design, Kaunas University of Technology, Studentu Str. 56, LT-51424 Kaunas, Lithuania

**Keywords:** AAO nanoporous membrane, two-step anodization method, high-frequency excitation method

## Abstract

In recent years, many research achievements in the field of anodic aluminum oxide (AAO) membranes can be observed. Nevertheless, it is still an interesting research topic due to its high versatility and applications in various fields, such as template-assisted methods, filtration, sensors, etc. Nowadays, miniaturization is an integral part of different technologies; therefore, research on micro- and nanosized elements is relevant in areas such as LEDs and OLEDs, solar cells, etc. To achieve an efficient mixing process of fluid flow in straight nanopores, acoustofluidic physics has attracted great interest in recent decades. Unfortunately, the renewal of the electrolyte concentration at the bottom of a pore is limited. Thus, excitation is used to improve fluid mixing along nanosized diameters. The effect of excitation by high-frequency vibrations on pore geometry is also investigated. In this study, theoretical simulations were performed. Using theoretical calculations, the acoustic pressure, acoustic velocity, and velocity magnitude were obtained at frequencies of 2, 20, and 40 kHz. Moreover, nanoporous AAO membranes were synthesized, and the influence of high-frequency vibrations on the geometry of the pores was determined. Using a high-frequency excitation of 20 kHz, the thickness of the AAO membrane increased by 17.8%. In addition, the thickness increased by 31.1% at 40 kHz and 33.3% at the resonant frequency of 40 kHz. Using high-frequency vibrations during the anodization process, the electrolyte inside the pores is mixed, and as a result, a higher oxide growth rate and a deeper structure can be achieved. On the other hand, to obtain pores of the same depth, the reaction can be performed in a shorter time.

## 1. Introduction

Porous anodic aluminum oxide (AAO) structures were first observed by Keller, Hunter, and Robinson in 1953 [1]. Then, a number of scientists, including Sir Nevill Francis Mott, who won the Nobel Prize in Physics in 1977, Hoar and Mott [2], and Dewald [3], proposed a mechanism for the formation of porous AAO. In 1990, Masuda, Tanaka, and Baba reported for the first time the fabrication process of highly ordered porous structured AAO membranes in oxalic acid [4]. Since then, new conditions have been discovered using different anodization regimes to obtain nanopores with different geometries. For example, anodization was performed using different types of acid electrolytes [5], changing experimental conditions such as the anodization temperature [6,7], the applied anodization potential [8], and the current [9], as well as the dependencies of the diameter of the pores and the distance between the pores, and the thickness of the AAO membranes on these parameters was discovered.

In recent years, researchers have made many achievements in the field of porous AAO membranes, so it is still an interesting research topic due to its great versatility and applications in various fields such as the fabrication of micro- and nanosized elements using the template-assistant approach [10,11,12,13], filtration [14,15,16,17,18], different types of sensors [19,20,21], tissue engineering [22], etc.

Moreover, looking at today’s technologies and their development, miniaturization is an integral “economic driver” of today [23,24,25,26,27], and research related to micro- and nanosized elements is relevant in areas such as mini-LEDs and OLEDs [28], solar cells [29,30], electrotherapy and drug delivery [31], sensors [32], etc. The growing potential of these technologies encourages the study of microchannels, which differ from conventional channels in their channel diameter [33,34,35,36]. Since flow regimes in small fluid volumes are often laminar, fluid heat exchange is limited, and it is likely that the fluid temperature increases along the length of the microchannel [37]. Therefore, to achieve an efficient and rapid fluid flow mixing process in straight microchannels, acoustofluidic physics has gained much interest in recent decades [38,39]. Surface acoustic waves (SAW) apply the effects of ultrasonic waves and have received a lot of attention from researchers due to their noninvasive nature and the advantages of efficient fluidic control [40]. Researchers have shown that mixing efficiency of the hot and cold fluid laminar flows can be improved by using acoustic streaming [41].

In terms of AAO pore geometry, the peculiarity of the AAO structure is the pore width-to-length ratio. Synthesized AAO membranes have been reported with pore diameters in the range of tens to several hundreds of nanometers, and the length can reach several tens of micrometers [13]. Therefore, the renewal of fluid at the bottom of the pore during anodization is limited. Given these points, the application of acoustofluidics in the fabrication of porous AAO membranes during the anodization process could be used to overcome this limitation. Moreover, the effect of excitation by high-frequency vibrations used during the anodization process on the pore geometry has not yet been widely investigated, because high-frequency vibrations usually are used for treatment processes before or after the anodization [42,43].

Taking into account different theoretical models [44] that explain AAO formation and provide insight into oxide dissolution at the oxide/electrolyte interface and ion migration under high-field conditions, as well as based on changes in findings on temperature and pH along the nanopore, it is important to ensure the mixing process and renewal of fluid flow along the length of the pore with nano- and microsized length. Moreover, considering the wide application of micro- and nanomaterials in various fields and the current research related to better mechanical strength, brittleness, and other improved properties of porous AAO membranes [45,46], the problem related to the temperature and pH value differences of the electrolyte flow inside the pore during the anodization process is analyzed in this paper. As the problem is relevant, and further research is needed, the solution to ensure a more uniform flow temperature and pH value in the pore of the AAO membrane when AAO is fabricated by the two-step anodization process is presented.

Therefore, in this article, two techniques (high-frequency excitation and chemical anodization) are combined to study fluid mixing inside the pores and to study the changes in the geometry of the AAO pores using high-frequency excitation during the well-known two-step anodization process using 0.3 M oxalic acid at a constant temperature of 5 °C and a constant voltage at 60 V. The structure of the paper is as follows. The theoretical simulation method, boundary conditions, equations used, as well as experimental AAO membrane fabrication technology are presented in Section 2. The theoretical simulations and the experimental results are presented in Section 3. The results of the influence of vibrations on the fluid mixing inside the pore and the influence of vibrations on the geometry of AAO membrane pores are presented. Conclusions are drawn in Section 4.

## 2. Materials and Methods

### 2.1. Simulation Method and Conditions of Vibration Process

**Figure 1:**
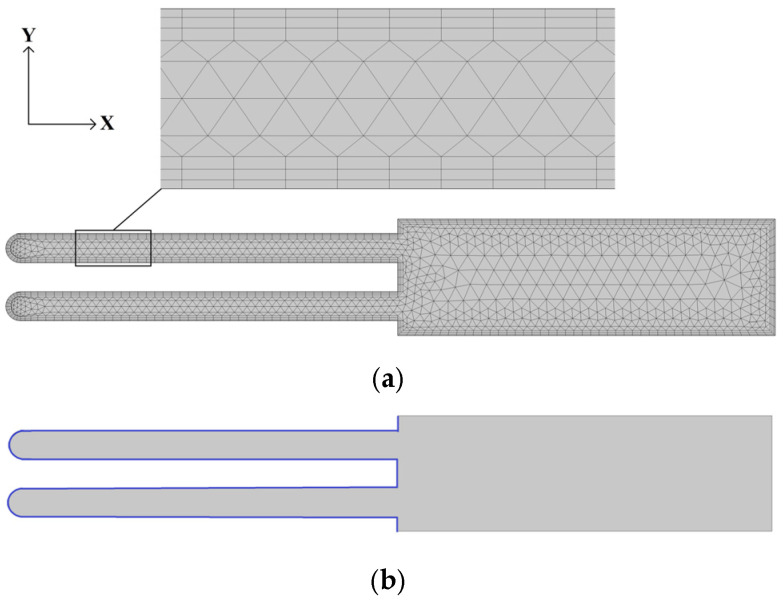
Simulation model. (**a**) Geometry and computational mesh for nanopores; (**b**) boundary conditions.

In this paper, the fluid flow inside nanopores was simulated using COMSOL Multiphysics 6.0 software. The numerical model consisted of two pores with a 105 nm diameter and an electrolyte container (part of the reactor volume). Two pores were chosen to determine the more realistic behavior of the electrolyte flow between the pores compared to the flow presented by a single pore. The model was meshed by finite tetrahedron elements with fixed support constraint boundary conditions. The computational mesh for the pores and the boundary conditions of the numerical model are presented in Figure 1.

Thus, the walls of the pores were solid surfaces with no-slip and isothermal boundary conditions. Therefore, the properties of the boundary layer were applied around the perimeter, where 3 layers were stretched (Figure 1a). The model consisted of 2764 elements with 320 boundary elements. Additionally, velocity and isothermal fortifications were used in the area marked in blue (Figure 1b). The velocity boundary conditions in the y direction were constrained, and movements in the x direction were unconstrained (periodic oscillation was selected). The entire domain was continuous and selected as a Thermoviscous Acoustics Model in which the equilibrium pressure was 1 atm, and the temperature was 5 °C. The properties of the electrolyte fluid were selected from Material Libraries in COMSOL Multiphysics 6.0. The parameters of the simulation model are presented in Table 1.

Since an acid electrolyte is used during anodization, the thermophysical properties of water have been used in the simulation. The thermophysical properties of fluid (water) are shown in Table 2.

In this study, two sets of governing equations were used to obtain the results. Based on the temporal and spatial scales, the acoustic velocity field was first calculated using the thermoviscous acoustics module in the frequency domain. Then, the streaming flow velocity field was calculated by applying the creeping flow module.

In the Thermoviscous Acoustics mathematical model, the following equations were used. Assuming small harmonic oscillations about the steady background properties, the dependent variables could be written as

p_t_ = p_1_ + p_b_ (1)

u_t_ = u_1_ + u_b_ (2)

T_t_ = T_1_ + T_b_ (3)

where p is the pressure, u is the velocity field, and T is the temperature. The prime variables (subscript 1) are the acoustic variables, and the variables accompanied by subscript b represent the background mean flow quantities.

In the Thermoviscous Acoustics interface, the continuity equation could be written as

p_t_ = p_1_ + p_b_ iω ρ_t_ + ∇ × (ρ_0_ u_t_) = 0 (4)

where ω is the frequency of actuation, ρ_0_ is the equilibrium density, u_t_ is the acoustic velocity field, and ρ_t_ is the density at temperature T_t_:

ρ_t_ = ρ_0_ (β_T_ p_T_ − α_p_ T_t_) (5)

β_T_ = (1/ρ_0_) − (γ/c^2^) (6)

α_p_ = (1/c) − ((c_p_ (γ − 1))/T_0_)^1/2^ (7)

where β_T_ is the isothermal compressibility coefficient, α_p_ is the coefficient of thermal expansion, c is the speed of sound in the fluid, and p_T_ is the equilibrium pressure. Equation (5) shows related variations in pressure, temperature, and density. The momentum equation could be written as

σ = −p_t_ I + µ (∇ u_t_ + (∇u_t_)^T^) − (2/3 µ − µ_B_) (∇ × u_t_) I (8)

where µ is the dynamic viscosity, and µ_B_ is the bulk viscosity. The right side of the equation indicates the divergence of the stress tensor.

In the frequency domain, multiplication with iω corresponds to differentiation with respect to time

iω ρ_0_ u_t_ = ∇ − σ (9)

Then, the energy conservation equation could be written as

ρ_0_ C_p_ (iω T_t_ + u_t_ × ∇ T_0_) − α_p_ T_0_ (iω p_t_ + u_t_ × ∇ p_0_) = ∇ × (k ∇ T_t_) + Q (10)

where C_p_ is the heat capacity at constant pressure, k is the thermal conductivity, α_p_ is the coefficient of thermal expansion (isobaric), and Q is a possible heat source.

Because creeping flow, also known as Stokes flow, could occur in the systems with small geometrical length scales, the following equations were used:

0 = ∇ × [−p 2 I + K] + F(11)

ρ ∇ × u_2_ = 0 (12)

K = µ (∇ u_2_ + (∇ u_2_)^T^) (13)

where ρ is density of the fluid, p is the fluid pressure field, u is velocity field of the fluid, and F is external force.

In the first study, frequencies of 2, 20, and 40 kHz were used. During the second study, the results obtained from the first simulation were used (each frequency was simulated separately).

### 2.2. Fabrication of AAO Nanoporous Membranes

**Figure 2:**
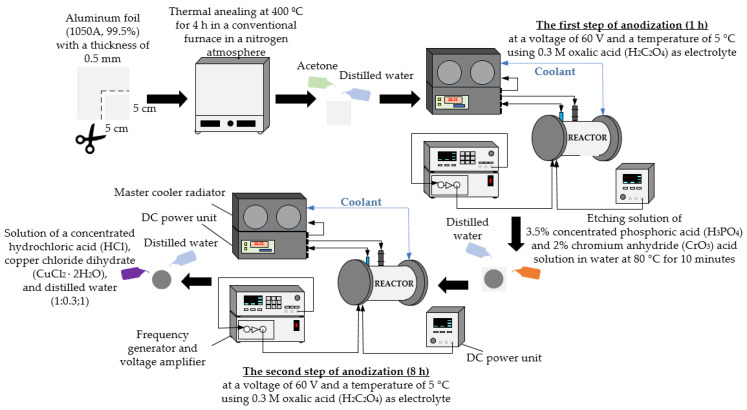
Illustration of the two-step anodization process.

AAO nanoporous membranes were fabricated using the two-step anodization process (Figure 2). A high-purity annealed aluminum foil (1050A, 99.5%) with a thickness of 0.5 mm was used for experiments. At first, aluminum foil was cut into 5 cm × 5 cm square specimens, which were annealed at 400 °C for 4 h under nitrogen ambient. Annealing was carried out in a conventional furnace. Then, the specimens were degreased in acetone and rinsed with distilled water. The prepared specimens were anodized in the electrochemical reactor. Anodization was performed at a voltage of 60 V and a temperature of 5 °C using 0.3 M oxalic acid (H_2_C_2_O_4_) as the electrolyte. The first anodization step lasted 1 h. Then, the synthesized oxide layer was chemically etched in a mixture of 3.5% concentrated phosphoric acid (H_3_PO_4_) and 2% chromium anhydride (CrO_3_) acid solution in water at 80 °C for 10 min. After being washed with distilled water, the specimens were placed in an electrochemical reactor, where the second step of anodization at 60 V and 5 °C temperature for 8 h was performed. Then, the aluminum layer from the AAO membrane was removed using a solution of a concentrated hydrochloric acid (HCl), copper chloride dihydrate (CuCl_2_ × 2H_2_O), and distilled water (1:0.3:1). Finally, the specimens were rinsed with distilled water and air-dried.

Under the same anodization conditions, AAO membranes were fabricated using high-frequency excitation during the first and second steps of anodization. The high-frequency oscillations were generated using a piezoceramic ring, which was excited by the signal from the frequency generator and the voltage amplifier. A piezoceramic ring was installed inside the reactor.

## 3. Results and Discussion

### 3.1. Influence of Vibration on Fluid Flow Inside the Pores

In this section, the simulations performed and the results obtained are described in order to verify the effect of high-frequency vibrations on the mixing process of the fluid flow inside membrane pores.

**Figure 3:**
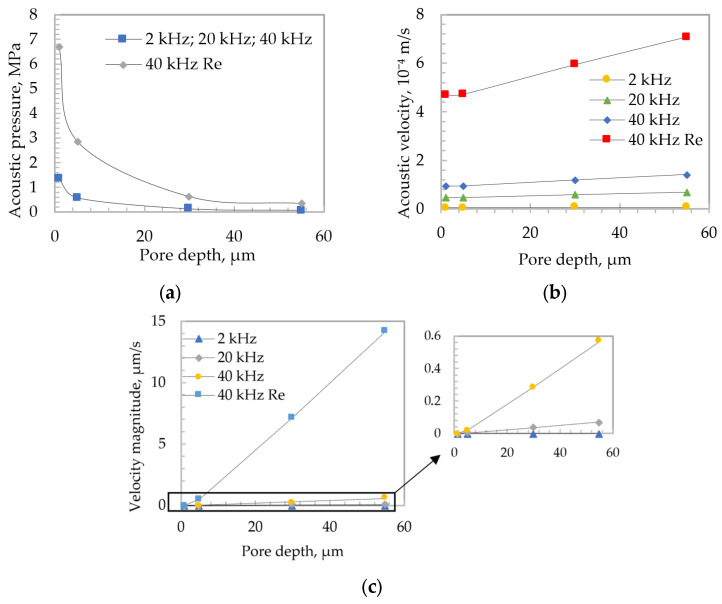
Comparative curves of different frequencies. (**a**) Acoustic pressure; (**b**) acoustic velocity; (**c**) velocity magnitude.

First, a line was drawn through the center of the entire length of the pore, which allowed for the collection of finite element method plot values. Three parameters were analyzed: total acoustic pressure, total acoustic velocity, and velocity magnitude. The simulations were performed at different frequencies of 2, 20, and 40 kHz. Considering the growth of oxide during the electrochemical process and the deepening of the pores, the simulations were also performed at different depths (1, 5, 30, and 55 µm) to evaluate how the total acoustic pressure, the total acoustic velocity, and velocity magnitude change with the change in the depth of the pores. At 1 and 5 µm depth, data were recorded at 4–5 nm, and at 30 and 55 µm depth, data were recorded at 7–8 nm. To evaluate changes in fluid flow at different excitation frequency values and pore depths, and to process large amounts of data, the calculation of mean values was used. Therefore, the dependencies of the variation in the average values of acoustic pressure, acoustic velocity, and velocity magnitude were obtained. The dependencies of different parameters on frequency and depth are presented in Figure 3.

The acoustic pressure decreased (Figure 3a) in all cases as the pore deepened. The highest acoustic pressure values were obtained at the resonant high-frequency excitation of 40 kHz. When evaluating the acoustic velocity (Figure 3b), the obtained results showed that the velocity slightly increased with the depth of the pore. The maximum value of the acoustic velocity was obtained at the resonant frequency of 40 kHz. When evaluating the velocity magnitude (Figure 3c), the maximum average values of the fluid flow velocity were also obtained at the resonant frequency of 40 kHz.

**Figure 4:**
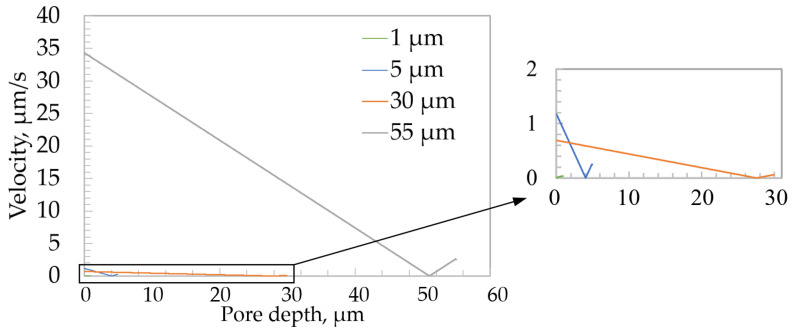
Comparative velocity curves of different pore depths.

Since the highest values were obtained at the resonant frequency of 40 kHz, the variation in the velocity value inside the pore at different pore depths is analyzed below. Comparative velocity curves are shown in Figure 4.

**Figure 5:**
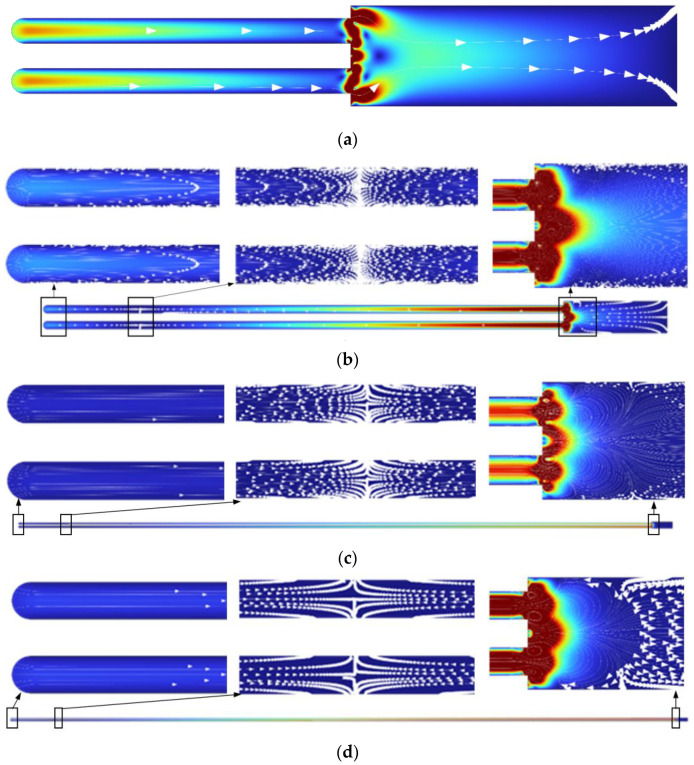
Fluid flow velocity contour at resonant frequency excitation of 40 kHz at different pore depths: (**a**) 1 µm; (**b**) 5 µm; (**c**) 30 µm; (**d**) 55 µm.

When evaluating the velocity dependence along the length of the pore, the velocity decreased uniformly as the pore deepened. In all the cases, close to the bottom of the pore, the velocity values decreased to zero and started to rise steadily until the fluid flow approached the bottom of the pore. At the point at which the flow velocity was zero, a barrier zone of unmixed flow appeared. At a depth of 1 µm, there was no unmixed barrier fluid flow, so the electrolyte could be intensively refreshed all the time. At a resonant frequency of 40 kHz, the velocity contours of fluid flow at different pore depths are presented in Figure 5.

As the pore depth increased, the speed and direction of the flow in the pore changed. At the beginning of the anodization process, when the depth of the pore was 1 µm, the electrolyte flowed throughout the length of the pore, and mixing also occurred at the bottom of the pore. As aluminum oxide formed, the pores became deeper. In the deeper pore, the barrier appeared, and the direction of fluid flow changed. Moreover, the results of the analysis showed that the highest speed was at the beginning of the pore. Inside the pore, the electrolyte flow rate was low (the mixing process was relatively slow) while the liquid outside (in the reactor) was intensively mixed. Such movement of the liquid during excitation was beneficial, because the electrolyte near the pore was intensively mixed with the entire reactor liquid, and the flow in the pore was constantly renewed. The results of the numerical simulations showed that mixing of the fluid flow inside the pore was ensured by using high-frequency excitation. Therefore, the electrolyte concentration inside the pore was also refreshed during the mixing process. Based on theoretical models of oxide growth, it was concluded that the pH value and the temperature change along the length of the pore. Therefore, high-frequency excitation during the anodization process could ensure a more uniform distribution of the electrolyte temperature and pH values along the entire length of the pores.

### 3.2. Influence of Vibration on Pore Geometry

After evaluating the theoretical fluid flow velocities and pressures inside the pore at different frequencies, experimental studies were carried out, during which it was possible to determine the effect of high-frequency vibrations on the pore geometry of AAO membrane. Since the most significant theoretical results were obtained at the frequency of 40 kHz, this frequency was further analyzed experimentally, and the frequency of 20 kHz was used for comparison.

Scanning electron microscopy (SEM) and energy dispersive spectroscopy (EDS) were used to determine the morphology and surface chemical composition of the nanopores of the AAO membranes. For analysis, the Hitachi S–3400N scanning electron microscope with an integrated Bruker energy dispersive X-ray spectroscopy (EDS) system was used. Using the “ImageJ” data-processing program, the diameters of the membrane pores, the distances between the pores, and the thickness of the obtained membranes were determined, and the results are presented in Table 3.

**Figure 6:**
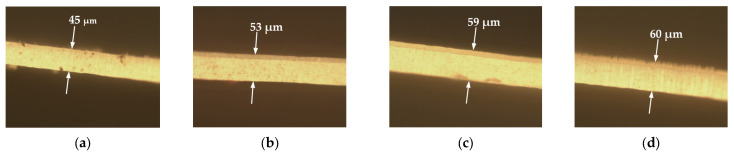
Images of the thickness of the AAO membrane when the membranes were fabricated under different anodization conditions: (**a**) no frequency excitation; (**b**) excitation at 20 kHz; (**c**) excitation at 40 kHz; (**d**) resonant frequency excitation at 40 kHz.

Using frequencies of 20 kHz and 40 kHz and the resonance frequency of 40 kHz, the pore diameters of the fabricated membrane were obtained. Respectively, they were 103 ± 10 nm, 105 ± 10 nm, and 105 ± 10 nm. Consequently, the interpore distances were 140 ± 10 nm, 140 ± 10 nm, and 145 ± 10 nm, and the thicknesses were 53 ± 0.5 µm, 59 ± 0.5 µm, and 60 ± 0.5 µm. It can be concluded that the pore diameter and the interpore distance remained unchanged under different anodization conditions. However, the thickness of the AAO membrane increased by 17.8% at 20 kHz. Furthermore, the thickness increased by 31.1% at 40 kHz and 33.3% at the resonant frequency of 40 kHz. The thickness of the AAO membrane was also measured using a microscope (Nikon Eclipse lv150, Tokyo, Japan). Images of the thickness of the AAO membrane are shown in Figure 6.

The chemical compositions of the nanoporous AAO membranes are shown in Table 4.

Analysis of EDS showed that two elements (aluminum and oxygen) predominated. It confirmed the formation of Al_2_O_3_. Due to amorphous aluminum oxide, two elements, sulfur and carbon, were identified as impurities. After the EDS analysis, it can be stated that the frequency did not affect the chemical composition of the membrane.

**Figure 7:**
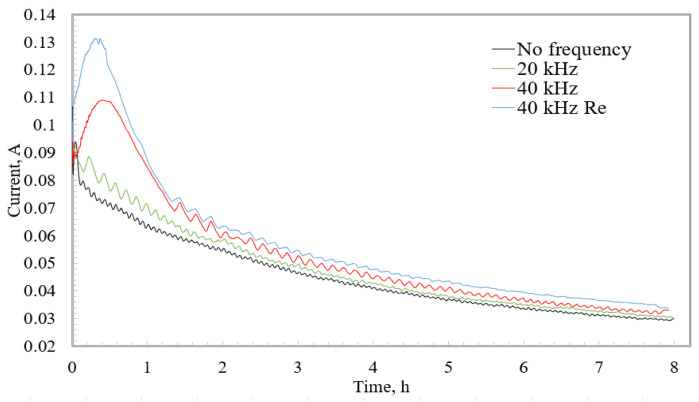
Current–time curves recorded during the second step of the anodization process.

Based on the analysis and comparisons of the complex “current-time” (I-t) curves, valuable conclusions about the established self-organization regime and ion migration, as well as new insights about the self-ordering oxide formation under different anodization conditions, were provided. Thus, during the second step of the anodization process, the anodization current was measured and observed. Current–time curves are presented in Figure 7.

In Figure 7, the curves of the current measurement in four different fabrication regimes were presented. The first curve, when anodization was performed without the use of high-frequency excitation, can be named the standard anodization curve. First, the curve was increased, and then the curve steadily decreased, revealing a slowing process. Slight current fluctuations were caused by changing the reactor temperature in the range of 4.5–6.0 °C. Using a high-frequency excitation of 20 kHz, it was possible to conclude from the increasing current curve that the anodization reactions intensified. This could be explained by faster ion migration. At a higher frequency of 40 kHz, in the first hour of anodization, the current was increased, which could be influenced by the increased frequency and the increased oscillating amplitude. At a resonant frequency of 40 kHz, the current was even more intense. As the current started to decrease, its value approached the standard current curve (when the frequency was not used), but the current remained slightly higher throughout the process. Such a change in the current could have been influenced by the liquid barrier that formed that is reviewed in the theoretical simulations. Although the refreshing process was not as intensive as at the beginning of the anodization, the electrolyte concentration was still updated due to the high frequency.

The experimental results and the current curves discussed above lead to the conclusion that the rate of the reaction and the rate of oxide formation are directly dependent on the concentration of the electrolyte. By renewing the electrolyte inside the pore, higher growth rates and deeper structure can be achieved. On the other hand, to obtain a pore of the same depth, the reaction can be performed in a shorter time when high-frequency vibrations are used.

## 4. Conclusions

A well-known two-step anodization process was proposed in which the excitation by high-frequency vibrations was used to ensure mixing of the fluid flow inside nanopores, thus ensuring a uniform temperature and pH value along the entire length of the pore. Further, the influence of the high-frequency vibrations on the geometry of AAO membrane pores was determined. The obtained results of the theoretical calculations showed that fluid flow inside the pores could be mixed more efficiently when high-frequency vibrations were used. The highest acoustic pressure, acoustic velocity, and velocity magnitude were obtained when the high-frequency excitation was applied at a resonant frequency of 40 kHz. Further, fabricated AAO membranes under different frequency conditions showed that the membrane thickness increased. The thickness of the AAO membrane increased by 17.8% at 20 kHz. The thickness also increased by 31.1% at 40 kHz and 33.3% at the resonant frequency of 40 kHz. The obtained experimental results agreed with the theoretical ones, which showed that at a resonant frequency of 40 kHz, due to the best mixing of the liquid flow in the pore, the reaction speed was the most intense, and therefore, the AAO membrane thickness was the highest during the same fabrication time. Moreover, it could be concluded that the pore diameter and the interpore distance remained unchanged using a frequency at 20 kHz and 40 kHz. Furthermore, after EDS analysis, it could be stated that the frequency did not affect the chemical composition of the AAO membrane. Current–time curves led to the conclusion that the rate of reaction and the rate of oxide formation were directly dependent on the concentration of the electrolyte in the pore. By renewing the electrolyte inside the pore, higher growth rates and a deeper structure could be achieved. On the contrary, in order to obtain a pore of the same depth, the reaction could be performed in a shorter time when high frequency vibrations were used.

## Figures and Tables

**Table 1:** Parameters of the model of fluid flow simulation in nanopores.

Parameter	Symbol	Inscription	Value	Units
Frequency	f0	2 [kHz]	2000	Hz
		20 [kHz]	20,000	
		40 [kHz]	40,000	
Ambient temperature	T0	5 [degC]	278.15	K
Ambient pressure	p0	1 [atm]	1.0133 × 10^5^	Pa
Angular frequency	omega0	2 × pi × f0	12,566	Hz
			1.257 × 10^5^	
			2.513 × 10^5^	
Mesh viscous penetration depth at f0	dvisc0	100 [um] × sqrt(100 [Hz]/f0)	2.236 × 10^−5^	m
			7.071 × 10^−6^	
			5.000 × 10^−6^	
Speed of sound in water	c0	1495.3 [m/s]	1495.3	m/s
Wavelength	lam0	c0/f0	0.747650	m
			0.074765	
			0.037383	
Wave number	k0	2 × pi/lam0	8.4039	1/m
			84.039	
			168.08	
Channel cross section width	W	105 [nm]	10.5 × 10^−8^	m
Channel cross-section height	H	1000 [nm]	1 × 10^−6^	m
		5000 [nm]	5 × 10^−6^	
		30,000 [nm]	30 × 10^−6^	
		55,000 [nm]	55 × 10^−6^	
Wall displacement	d0	1 [nm]	1 × 10^−9^	m
		5 [nm]	5 × 10^−9^	

**Table 2:** Thermophysical properties of fluid (water).

Fluid	Density, kg/m^3^	Dynamic Viscosity, Pa·s	Bulk Viscosity, Pa·s	Ratio of Specific Heats	Heat Capacity at Constant Pressure, J/(kg·K)	Thermal Conductivity, W/(m·K)	Speed of Sound, m/s
Water	1000	0.0018	0.005	1	4200	0.56	1400

**Table 3:** Pore diameter, interpore distance, and thickness of AAO nanoporous membranes.

Parameter	Pore Diameter (nm)	Interpore Distance (nm)	Thickness (µm)
No excitation	104 ± 10	143 ± 10	45 ± 0.5
Excitation frequency 20 kHz	103 ± 10	140 ± 10	53 ± 0.5
Excitation frequency 40 kHz	105 ± 10	140 ± 10	59 ± 0.5
Resonant excitation frequency 40 kHz	105 ± 10	145 ± 10	60 ± 0.5

**Table 4:** Chemical composition of AAO nanoporous membranes.

		Element			
		Aluminum	Oxygen	Carbon	Sulfur
No excitation	Atomic concentration, at%	35.39	63.15	1.18	0.28
	Error, %	2.3	6.9	0.3	0.1
Excitation frequency 20 kHz	Atomic concentration, at%	31.92	64.78	2.90	0.41
	Error, %	2.3	7.9	0.6	0.1
Excitation frequency 40 kHz	Atomic concentration, at%	31.44	66.02	2.11	0.43
	Error, %	2.2	7.4	0.4	0.1
Resonant excitation frequency 40 kHz	Atomic concentration, at%	32.38	66.73	0.23	0.66
	Error, %	2.1	7.3	0.3	0.1

## Data Availability

Not applicable.
